# Engineering CRISPR/Cas9 to mitigate abundant host contamination for 16S rRNA gene-based amplicon sequencing

**DOI:** 10.1186/s40168-020-00859-0

**Published:** 2020-06-03

**Authors:** Luyang Song, Kabin Xie

**Affiliations:** National Key Laboratory of Crop Genetic Improvement and Hubei Key Laboratory of Plant Pathology, Huazhong Agricultural University, No.1 Shizishan Street, Hongshan District, Wuhan, 430070 China

**Keywords:** 16S-seq, CRISPR-Cas, Host contamination, Microbiome, Plant

## Abstract

**Background:**

High-throughput sequencing of bacterial 16S rRNA gene (16S-seq) is a useful and common method for studying bacterial community structures. However, contamination of the 16S rRNA genes from the mitochondrion and plastid hinders the sensitive bacterial 16S-seq in plant microbiota profiling, especially for some plant species such as rice. To date, efficiently mitigating such host contamination without a bias is challenging in 16S rRNA gene-based amplicon sequencing.

**Results:**

We developed Cas-16S-seq method to reduce abundant host contamination for plant microbiota profiling. This method utilizes the Cas9 nuclease and specific guide RNA (gRNA) to cut 16S rRNA targets during library construction, thereby removing host contamination in 16S-seq. We used rice as an example to validate the feasibility and effectiveness of Cas-16S-seq. We established a bioinformatics pipeline to design gRNAs that specifically target rice 16S rRNA genes without bacterial 16S rRNA off-targets. We compared the effectiveness of Cas-16S-seq with that of the commonly used 16S-seq method for artificially mixed 16S rRNA gene communities, paddy soil, rice root, and phyllosphere samples. The results showed that Cas-16S-seq substantially reduces the fraction of rice 16S rRNA gene sequences from 63.2 to 2.9% in root samples and from 99.4 to 11.6% in phyllosphere samples on average. Consequently, Cas-16S-seq detected more bacterial species than the 16S-seq in plant samples. Importantly, when analyzing soil samples, Cas-16S-seq and 16S-seq showed almost identical bacterial communities, suggesting that Cas-16S-seq with host-specific gRNAs that we designed has no off-target in rice microbiota profiling.

**Conclusion:**

Our Cas-16S-seq can efficiently remove abundant host contamination without a bias for 16S rRNA gene-based amplicon sequencing, thereby enabling deeper bacterial community profiling with a low cost and high flexibility. Thus, we anticipate that this method would be a useful tool for plant microbiomics.

Video Abstract

## Background

Microbiota play essential roles in animal and plant health [1, 2]. A deeper understanding of plant microbiota would provide new strategies to regulate plant growth, enhance crop stress tolerance, and reduce pathogenesis for sustainable agriculture [3–5]. Massive parallel sequencing of marker genes, such as the 16S small subunit ribosomal RNA gene (16S rRNA gene), is a useful culture-independent approach to study the microbiome [6–9]. 16S-seq uses broad-range universal primers to amplify one or more hypervariable regions (V1-V9) of 16S rRNA gene and thereby infer the assemblage and potential functionality of microbiota [10]. These universal primers also target the eukaryotic mitochondria and plastid 16S rRNA genes, which are derived from prokaryotic ancestors. As a result, the plastid and mitochondrial sequences could account for up to 99% of the reads in 16S-seq data when analyzing microbial communities derived from plant samples [11, 12]. Such contamination of host 16S rRNA genes drastically impairs plant microbiome studies.

Eliminating abundant host 16S rRNA genes mixed with numerous bacterial homologs from amplification in PCR is challenging. PCR clamping, which was originally designed to suppress the amplification of a particular allele during PCR [13], is able to substantially mitigate host 16S rRNA gene contamination for 16S-seq [13, 14]. PCR clamping utilizes peptide nucleic acid (PNA) and locked nucleic acid (LNA) oligos to prevent PCR elongation or primer binding of target DNA. Few host-specific PNA [11, 14–16] and LNA [17, 18] oligos have been designed to specifically suppress the amplification of plastid and mitochondrial 16S rRNA genes. However, a recent study found that the PNA oligo also blocked certain bacterial sequences from PCR amplification, thereby introducing a significant bias in microbiota profiling [19]. Thus, an efficient and non-biased method that specifically eliminates abundant host 16S rRNA genes in 16S-seq is awaited for characterization of plant microbiomes.

CRISPR (clustered regularly interspaced short palindromic repeat)-Cas technologies provide powerful platforms to precisely manipulate DNA. CRISPR-Cas is an adaptive immune system in bacteria and archaea and has become a revolutionary tool for genome editing in recent years [20]. Among different CRISPR-Cas systems, Cas9 from *Streptococcus pyogenes* is broadly used in genome editing [20, 21] and enables many innovative applications in molecular biology. Cas9 is directed to the DNA target by a single-guide RNA (gRNA) and specifically cleaves the site [22, 23]. By replacing the 20 nucleotide guide sequence in the 5′-end of gRNA, Cas9 can be reprogrammed to cleave any DNA sequence of 5′-N20-NGG-3′ (N indicates any nucleotide), in which N20 is identical to the gRNA guide sequence and NGG is the indispensable protospacer-adjacent-motif (PAM) for Cas9 activity. In addition to genome editing, CRISPR/Cas9 has been engineered to remove unwanted DNA sequences or to enrich desired DNA fragments in next-generation sequencing (NGS). For example, STR-seq [24] and CRISPR-Cap [25] methods employ Cas9 to enrich desired genomic DNA regions for deep sequencing. In addition, Cas9 was programmed to cut specific DNA targets, thereby removing unwanted DNA molecules for transcriptomics. Previous studies suggested that Cas9 could efficiently remove rRNA fractions in RNA-seq [26] and ATAC-seq [27]. These findings inspired us to address the challenge of eliminating host contamination in 16S-seq using CRISPR/Cas9.

**Fig. 1:**
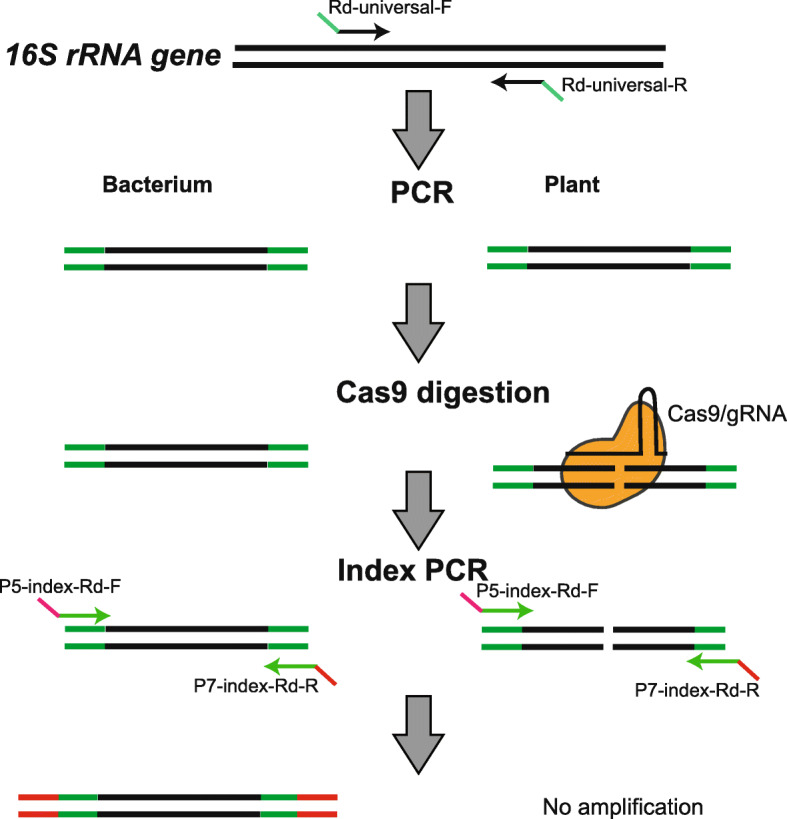
Schematic diagram of Cas-16S-seq. Cas-16S-seq is based on the regular 16S-seq procedure using two-step PCR to amplify and index 16S rRNA genes. In the first PCR, universal primers with adaptors (Rd-universal-F/R) are used to amplify the variable regions of 16S rRNA genes. Then, Cas9 and specific gRNA is used to cleave the host 16S rRNA genes in the PCR product. In the second index PCR, primers containing index and Illumina sequencing adaptors (P5-index-Rd-F and P7-index-Rd-R) are used to amplify the digested products and obtain the final amplicon library. The host 16S rRNA gene fragments are not amplified in the second PCR and are therefore removed in the final 16S-seq data.

In this study, we developed a Cas-16S-seq method to mitigate host contamination for 16S rRNA gene-based amplicon sequencing. This method uses Cas9 and specific gRNA to exclusively cut host 16S rRNA genes and subsequently enrich bacterial sequences during preparation of 16S-seq amplicon libraries (Fig. 1). Using rice plant as an example, we established a bioinformatics pipeline to design specific gRNAs that discriminate rice 16S rRNA genes from millions of prokaryotic 16S rRNA genes. After comparisons of Cas-16S-seq and regular 16S-seq using artificially mixed 16S rRNA gene communities, paddy soil, rice root, and phyllosphere samples, we demonstrated that Cas-16S-seq is highly efficient and specific to remove host 16S rRNA genes without a bias. Given its efficiency, simplicity, and low cost, we anticipate that Cas-16S-seq would be broadly used to improve the performance of 16S-seq to study the plant microbiome.

## Results

### The workflow for Cas-16S-seq method

Abundant host plastid or mitochondrial 16S rRNA gene sequences mask plant microbiomes in 16S-seq profiling [14]. We used rice as an example to tackle this challenge. Sequence analysis showed that the universal primers for 16S-seq, including 27F, 338R, 357F, 515F, 806R, U519F, U806R, 799F, 804F, 907R, 926F, 1114F, 1193R, 1380F, 1392R, and 1492R, perfectly match at least one of rice 16S rRNA genes derived from mitochondrion or chloroplast (Additional file 1: Figure S1). We tested four sets of universal 16S primer pairs that are often used in plant microbiome studies, including 515F-806R [12, 16, 28–30], 27F-338R [31], 799F-1193R [32, 33], and 1114F-1392R [11], for PCR using rice DNA. We found robust amplification of rice mitochondrion (515F-806R, 799F-1193R) and chloroplast (27F-338R, 515F-806R, 1114F-1392R) 16S rRNA genes (Additional file 1: Figure S2). The annealing sites of these universal primers are located within the most conserved regions of 16S rRNA genes, which allows a broad coverage of bacterial sequences. Therefore, any alteration in primer sequences would affect their coverage and introduce significant bias in analysis [34]. To address this problem, we developed Cas-16S-seq method, which employs an RNA-programmable nuclease Cas9 to specifically eliminate host contamination from 16S-seq amplicons.

The workflow for Cas-16S-seq is shown in Fig. 1. Cas-16S-seq uses the same two-step PCR procedure as the common 16S-seq method [35]. The first PCR uses universal primers with the appropriate adaptor to amplify variable regions of 16S rRNA gene. The second index PCR amplifies the products of the 1st round PCR with primers containing Illumina sequencing primers (P5 and P7) and indices (i5 and i7). In Cas-16S-seq, Cas9 and host 16S rRNA-specific gRNA are introduced to digest the first round of PCR products. The gRNA was designed for the host 16S rRNA genes without targeting bacterial sequences (Fig. 1). The cleaved host 16S rRNA fragments would not be amplified in the second PCR and thus do not appear in the final sequencing library (Fig. 1). In comparison to the common 16S-seq, Cas9-16S-seq only includes one additional step (Cas9/gRNA treatment) after the first PCR and before the second PCR; thus, it can be easily integrated into the current workflow of 16S rRNA gene-based amplicon sequencing.

### In silico design of gRNAs specifically targeting rice 16S rRNA genes

**Fig. 2:**
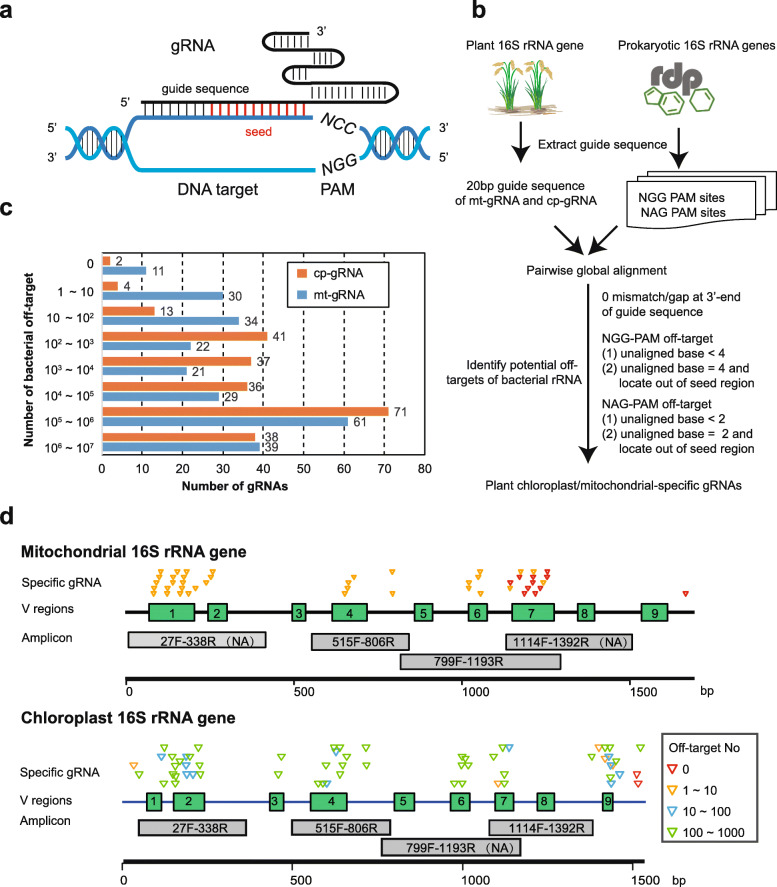
In silico design of host 16S rRNA gene-specific gRNAs for Cas-16S-seq in rice. **a** The schematics of targeted DNA cleavage using Cas9 and gRNA. The 20 bp DNA target is paired with the gRNA guide sequence. A protospacer-adjacent motif (PAM, 5′-NGG-3′), which is indispensable for Cas9 binding, immediately follows the paired region. Red indicates the seed region that is less tolerance to mismatches for Cas9 binding. **b** The flowchart of the bioinformatics analysis procedure to evaluate gRNA specificities. The guide sequences of gRNA that target rice mitochondrial and chloroplast 16S rRNA gene (mt-gRNA and cp-gRNA) were extracted according to the requirements for Cas9/gRNA binding (Fig. 2a). Then, these guide sequences were aligned to prokaryotic 16S rRNA genes in the RDP database. Both NGG PAM and NAG PAM off-target sites in RDP-rRNA were considered here, although Cas9 weakly recognized NAG-PAM. The RDP-rRNA off-targets of each gRNA were identified using the criteria shown in the flowchart (see details in the “Methods” section). Finally, the total number of bacterial off-targets for each gRNA was used to rank the gRNA specificities (see Additional file 2: Table S1 and S2). **c** Number of mt-gRNAs and cp-gRNAs with different specificity ranks according to the number of RDP-rRNA off-targets. **d** Distribution of most specific mt-gRNA (< 10 off-targets) and cp-gRNA (< 1000 off-targets). The green boxes indicate hypervariable regions (V1–V9) of 16S rRNA gene. Gray boxes indicate the region of four 16S rRNA amplicons. The triangles indicate the gRNA position and are colored according to the number of RDP-rRNA off-targets. NA, not amplified

The feasibility of Cas9-16S-seq would rely on high specificity of gRNAs that can distinguish host and bacterial 16S rRNA genes. Cas9/gRNA can efficiently cleave DNA that perfectly matches gRNA but also cleave partially matched off-targets with reduced activity (Fig. 2a). Extensive studies on Cas9-mediated genome editing in recent years illuminated the targeting rules for CRISPR/Cas9 [36–38]; thus, the potential off-targets of a given gRNA could be predicted according to these rules. In addition, a comprehensive collection of high-quality prokaryotic 16S rRNA gene sequences is available from public databases, including RDP [39], SILVA [40], and GreenGenes [41]. These databases have been used as references for metagenomic analysis and can be used to design gRNAs for Cas-16S-seq. To design rice 16S rRNA-specific gRNAs, we modified the programs used in CRISPR-PLANT, which enable genome-wide identification of off-targets of gRNAs [42, 43], to analyze prokaryotic 16S rRNA gene sequences (Fig. 2b). According to the PAM (5′-NGG-3′) required for Cas9 targeting, 243 and 247 gRNAs are available to target rice chloroplast and mitochondrial 16S rRNA genes, respectively (referred to as cp-gRNA and mt-gRNA). The 20 nt guide sequences of these gRNAs were used to search RDP database (release 11.5), which contain 3,356,809 aligned and high-quality 16S rRNA gene sequences (RDP-rRNA). Because Cas9 also weakly recognizes 5′-NAG-3′ as a PAM [38], we considered both the NGG-PAM and NAG-PAM sites in RDP-rRNA. The potential bacterial targets of mt-gRNA and cp-gRNA were identified according to sequence similarities as well as mismatch/gap positions in a gRNA-DNA alignment (Fig. 2b, see details in the “Methods” section). We also excluded 71,940 chloroplast 16S rRNA gene sequences from RDP-rRNA dataset for cp-gRNA specificity assessment. These gRNAs have very diverse range of RDP-rRNA off-target number (Fig. 2c). For instance, two cp-gRNAs and eleven mt-gRNAs had no off-target while eight mt-gRNAs and ten cp-gRNAs, whose guide sequences are overlapped with universal primers, had off-targets with 70–78% RDP-rRNA sequences (Fig. 2c, Additional file 2: Table S1 and S2). We ranked the gRNA specificity by counting the total number of potential bacterial off-targets in the RDP-rRNA dataset and selected 105 mt-gRNAs and 83 cp-gRNAs with less than 3300 (0.1% of RDP-rRNA) RDP-rRNA off-targets as the candidates of specific gRNAs for Cas-16S-seq (Additional file 2: Table S1 and S2).

We then examined the target sites of rice-specific mt-gRNA and cp-gRNAs in four different 16S rRNA amplicons. Of note, PCR using primers 27F-338R and 1114F-1392R did not amplify mitochondrial 16S rRNA gene, while 799F-1193R did not amplify chloroplast 16S rRNA gene (Additional file 1: Figure S1 and S2). Hence, rice-specific cp-gRNAs are required for 27F-338R (V1-V2), 515F-806R (V4), and 1114F-1392R (V7-V8) amplicons, while mt-gRNAs are required for 515F-806R (V4) and 799F-1193R (V5-V7) amplicons. As shown in Fig. 2d, the specific mt-gRNAs and cp-gRNAs we selected are not evenly distributed in rice mitochondrial and chloroplast 16S rRNA genes. Ten of eleven most specific mt-gRNAs with no off-targets (Fig. 2c) are located in the V7 region of mitochondrial 16S rRNA gene (Fig. 2d). Other host-specific mt-gRNAs with fewer than 10 RDP-rRNA off-targets locate in the V1, V2, V4, V6, and V7 regions. Although the number of specific cp-gRNAs targeting V1–V9 regions is less than that of mt-gRNA (Fig. 2c, d), we also found cp-gRNAs with less than 1000 (0.03%) RDP-rDNA off-targets for all V regions except V5 and V8 (Fig. 2d, Additional file 2: Table S2). These gRNAs have minimized risk to off-target bacterial 16S rRNA gene sequences in rice samples and could be used to target the short amplicons or full-length rice 16S rRNA genes for Cas-16S-seq.

### Cas9 and mt-gRNA efficiently eliminate the host 16S rRNA genes in mock samples

To examine the cleavage efficiency of Cas9, we tested twelve mt-gRNAs and five cp-gRNAs using purified rice 16S rRNA gene amplicons (Additional file 1: Figure S3). Thirteen gRNAs showed 90–100% efficiencies, while four gRNAs showed moderate efficiencies (65–84%) for Cas9-mediated cleavage of rice 16S rRNA gene amplicons (Additional file 1: Figure S3). These data are consistent with different efficiencies of gRNAs in the Cas9 genome editing [44]. Among the five gRNAs that exhibited 100% cleavage efficiencies, we selected mt-gRNA1048, mt-gRNA1171, and mt-gRNA1196 for Cas-16S-seq. All three gRNAs target the 799F-1193R amplicon which importantly does not contain the chloroplast 16S rRNA gene. mt-gRNA1171 and mt-gRNA1196 do not have off-targets in RDP-rRNA, while mt-gRNA1048 has ten RDP-rRNA off-targets based on our prediction (Additional file 2: Table S1).

**Fig. 3:**
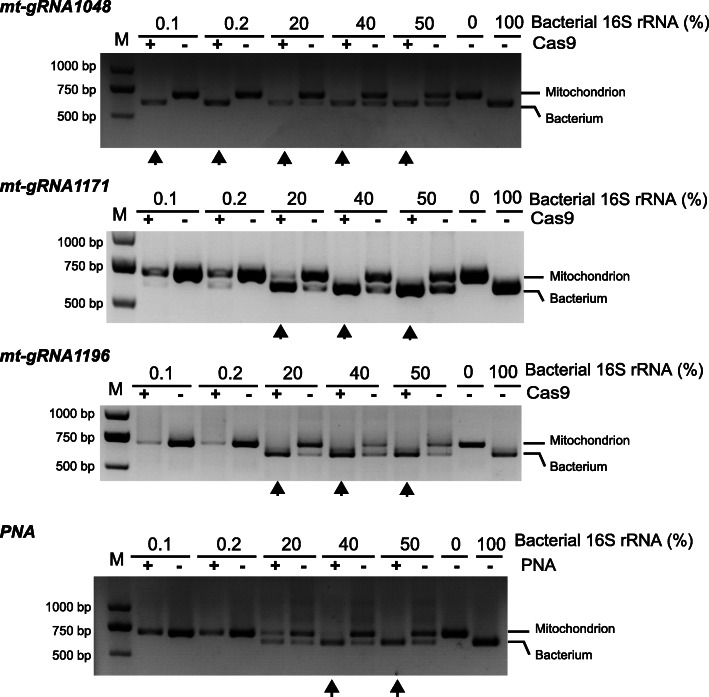
Cas-16S-seq efficiently removed the rice mitochondrial 16S rRNA gene fractions in mock communities. Three gRNAs (mt-gRNA1048, mt-gRNA1171, and mt-gRNA1196) were used to evaluate the performance of Cas-16S-seq. Mock samples containing 0%, 0.1%, 0.2%, 20%, 40%, 50%, and 100% bacterial 16S rRNA gene fragments were treated with Cas9/gRNA and then amplified by PCR. The depletion efficiency of host 16S rRNA genes was estimated by gel electrophoresis since the mitochondrial PCR product is approximately 84 bp larger than that of bacteria. The PNA PCR clamping method was also performed using the same mock samples. Arrows indicate the treatments by which the mitochondrial 16S rRNA gene fraction was efficiently eliminated. M, DNA marker; bp, base pair

We then tested the effectiveness of Cas-16S-seq using mock communities that were prepared by mixing DNA fragments from rice mitochondrial and soil bacterial (NCBI accession number: AB658673) 799F-1193R sequences at different ratios. These mock samples contained 0%, 0.1%, 0.2%, 20%, 40%, 50%, and 100% bacterial 16S rRNA gene sequences. Because the bacterial 799F-1193R amplicon is 84 bp shorter than the of rice mitochondrial amplicon (Additional file 1: Figure S1), the relative abundance of mitochondrial 16S rRNA in PCR products could be readily determined by gel electrophoresis. The results showed that the 16S-seq amplicons included rice mitochondrial 16S rRNA fragments with their intensities reflecting the amount of the input rice mitochondrial DNA (Fig. 3). In sharp contrast, amplification of rice 16S rRNA was greatly reduced in Cas-16S-seq (Fig. 3). Strikingly, rice 16S rRNA gene was not detected in mock sample containing only 0.1% bacterial DNA and 99.9% rice DNA in Cas-16S-seq with mt-gRNA1048. We also tested the PCR clamping method using a PNA oligo targeting rice mitochondrial 16S rRNA gene. We observed that the PNA PCR clamping method efficiently suppressed the amplification of its target in mock samples containing 40% and 50% bacterial 16S rRNA gene fragments. However, the PNA PCR clamping did not enrich bacterial sequences in mock samples containing 0.1–20% bacterial 16S rRNA fraction (Fig. 3). Thus, Cas-16S-seq is more efficient than the PNA PCR clamping in eliminating the contamination of rice mitochondrial 16S rRNA gene when low amount of bacterial rRNA exists, which resembles natural plant microbiota samples (Fig. 3).

### Evaluation of Cas-16S-seq using paddy soil samples

**Fig. 4:**
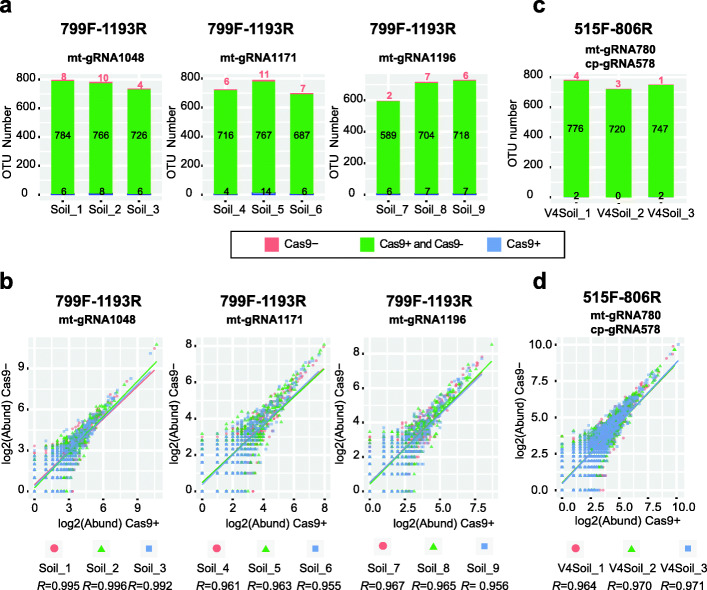
Evaluation of Cas-16S-seq using paddy soil samples. Three biological replicates per gRNA were performed to compare the Cas-16S-seq (Cas9+) and regular 16S-seq (Cas9−) for 799F-1193R and 515F-806R amplicons. The 799F-1193R amplicons were digested using mt-gRNA1048, mt-gRNA1171, or mt-gRNA1196 (**a**, **b**); the 515F-806R amplicons were digested with two gRNAs (mt-gRNA780 and cp-gRNA578, **c**, **d**). The OTU tables were rarefied to 16,000 sequences. **a**, **c** The observed OTUs (average relative abundance per sample > 2e−4) were highly consistent between Cas-16S-seq and regular 16S-seq results for each sample. The boxplots show the number of OTUs detected by both methods (Cas9+ & Cas9−, colored in green), by Cas-16S-seq only (Cas9+, colored in blue) and by regular 16S-seq only (Cas9−, colored in red). **b**, **d** The abundances of observed OTUs are highly consistent between Cas-16S-seq (log_2_(Abund) Ca9+) and regular 16S-seq (log_2_(Abund) Cas9−) data in all comparisons. The OTU abundances were log_2_ transformed. The correlation coefficients (*R*) for each comparison are shown at the bottom. The different biological replicates are indicated by color

Next, we asked whether Cas-16S-seq using mt-gRNA1048, mt-gRNA1171, and mt-gRNA1196 introduced bias when analyzing soil bacterial communities. To this end, we prepared amplicon libraries using Cas-16S-seq (Cas9+) and regular 16S-seq (Cas9-). Three biological replicates were performed for each mt-gRNA. These 799F-1193R amplicon libraries were sequenced by Illumina 2 × 250 bp paired-end sequencing, and a total of 785,371 sequences were obtained after quality filtration, dereplication, and denoising. These sequences were clustered into operation taxonomic units (OTUs) with 100% similarities. The bacterial diversities were compared after rarefying OTU table to 16,000 reads per sample (Additional file 3: Table S3). Cas-16S-seq and 16S-seq detected a comparative number of OTUs with relative abundance > 0.02% for all samples (Fig. 4a and Additional file 3: Table S3, paired Student’s *t* test, *p* = 0.67). Among these OTUs, 96.8–98.7% of them were detected by both methods (Fig. 4a). Only few low abundant OTUs that were only detected by one method were likely due to artefacts during PCR or sequencing. We did not detect significant differences in bacterial diversities (Shannon and Simpson indices) between Cas-16S-seq and 16S-seq (paired Student’s *t* test, Shannon-*p* = 0.10 and Simpson-*p* = 0.86; Additional file 3: Table S3). Importantly, the abundance of these OTUs was highly correlated between the Cas9+ and Cas9− data (Pearson’s correlation coefficient, *R* = 0.955–0.996, Fig. 4b). We also used DESeq2 [45] to test the difference on OTU abundances between the two methods. Only one OTU (OTU_1), which is rice mitochondrial 16S rRNA gene, was depleted in the Cas-16S-seq data (Wald test, *p* < 0.05, Additional file 3: Table S4). This is not surprising because the paddy soil samples have 0.048–0.26% rice mitochondrial 16S rRNA gene sequences, and these sequences were removed in Cas-16S-seq (Additional file 3: Table S3). The high consistency of bacterial profiles between Cas-16S-seq and regular 16S-seq indicated that Cas-16S-seq does not introduce bias to soil microbiota profiling.

### Cas-16S-seq efficiently removes host contamination from root samples

**Fig. 5:**
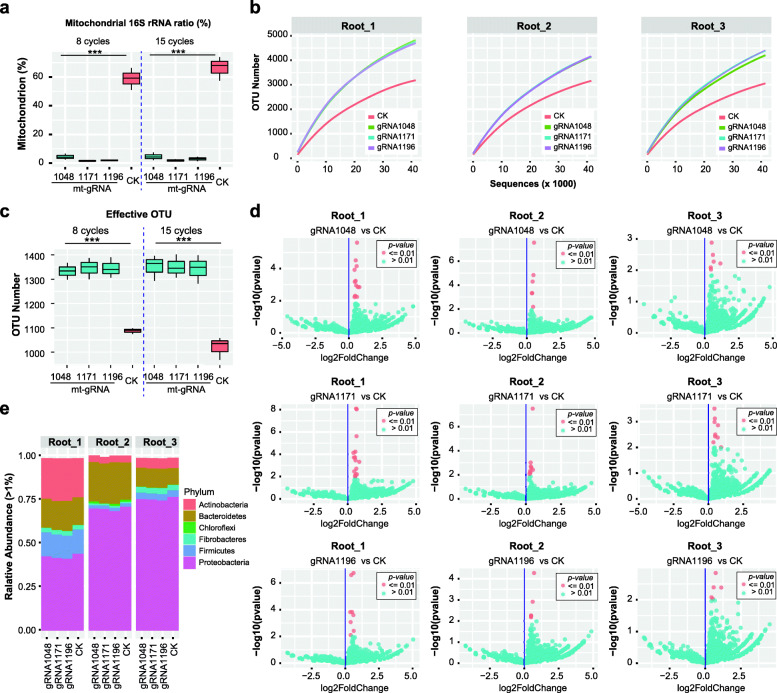
Cas-16S-seq efficiently and specifically mitigated mitochondrial 16S rRNA gene fractions in rice root microbiota profiling. Three biological replicates (Root_1–Root_3) and two technique replicates (8 and 15 index PCR cycles) were performed for each mt-gRNA treatment and control. The OTU table was rarefied to 41,500 reads per sample. **a** Comparison of rice mitochondrial 16S rRNA gene contents in Cas-16S-seq and regular 16S-seq (CK) sequencing results. The host 16S rRNA gene was drastically reduced after Cas9 treatment with 3 mt-gRNAs. ***ANOVA, *p* = 5e−16. **b** Rarefaction curve of Cas-16S-seq and regular 16S-seq data. The rarefaction analysis result using the data from one technique repeat (8 cycles) is shown here. **c** Cas-16S-seq observed more OTUs with average count per sample > 1 than regular 16S-seq. ***ANOVA, *p* = 4.35e−10 (see also Additional file 4: Table S6). **d** Analysis of differential bacterial OTUs between Cas-16S-seq and regular 16S-seq (CK). No OTU was significantly reduced in relative abundance after Cas9/gRNA treatments in all comparisons (Cas-16S-seq vs regular 16S-seq). In contrast, 29 OTUs were significantly increased in the Cas-16S-seq data (indicated by red dots; Wald test, *p* < 0.01; see also Additional file 4: Table S6). **e** Composition of bacterial communities at the phylum level. Bacterial phyla with relative abundances > 1% were shown here

We then explored the performance of the Cas9-16S-seq method to remove abundant host contamination in 799F-1193R amplicons using rice root samples which include rhizoplane and endosphere compartments. Again, we compared Cas-16S-seq and regular 16S-seq libraries from three rice root samples. To account for artefacts during PCR and sequencing, two technical replicates were performed with different index PCR cycles (8 and 15 cycles) for each treatment (Cas9+ vs Cas9−). The gel electrophoresis of 799F-1193R amplicons showed that the rice 16S rRNA genes were efficiently removed using Cas-16S-seq (Additional file 1: Figure S4). Illumina paired-end sequencing results also indicated that the rice mitochondrial 16S rRNA gene fraction in the Cas-16S-seq amplicon (2.9 ± 1.5%) was 21 times less than in the 16S-seq amplicon (63.2 ± 8.4%) on average (Fig. 5a, Additional file 4: Table S5; ANOVA, *F* test, *p* = 5e − 16). The performance of three mt-gRNAs also showed subtle but statistically significant difference (ANOVA, *F* test, *p* = 0.016). Among three gRNAs, mt-gRNA1048 (4.7 ± 2.3%) amplicons had slightly more rice 16S rRNA gene fraction than mt-gRNA1171 (1.7 ± 0.7%) and mt-gRNA1196 (2.4 ± 1.2%) amplicons. In comparison with 16S-seq amplicons, the rates of chimeras were slightly increased in Cas-16S-seq amplicons in both technical replicates but the difference was not statistically significant (ANOVA, *F* test, *p* = 0.173 and 0.174 for technical replicate with 8 and 15 PCR cycles, respectively; Additional file 1: Figure S5), implying that the cleaved host 16S rRNA gene fragment has a negligible effect on chimera formation during PCR amplification. In summary, the abundant host contamination in root samples was efficiently removed using the Cas-16S-seq method.

We compared Cas-16S-seq and regular 16S-seq for microbial profiling. The rarefaction curve indicates that Cas-16S-seq using either mt-gRNA produced higher bacterial richness than 16S-seq (Fig. 5b). Since the abundant host contamination was removed, Cas-16S-seq detected an average of 1.3 times more OTUs than 16S-seq (ANOVA, *F* test, *p* = 4.35e−10, Fig. 5c). Consequently, bacterial community compositions in Cas-16S-seq are significantly different from those in 16S-seq (PERMANOVA, *R*^*2*^ = 0.15, *p* < 0.001; Additional file 1: Figure S6). We tested the alpha diversities (Shannon and Simpson indices) after removing the mitochondrial sequences and detected no significant difference between the two methods (ANOVA, *F* test, Shannon *p* = 0.856, Simpson *p* = 0.96; Additional file 4: Table S5). To determine whether any bacterial 16S rRNA gene was removed specifically in Cas-16S-seq as compared with 16S-seq, we analyzed the differential OTUs between two methods using DESeq2. We did not detect any bacterial OTU with significantly reduced relative abundance after Cas9/gRNA treatment (Fig. 5d and Additional file 4: Table S6), suggesting that we did not eliminate particular bacterial 16S rRNA genes in Cas-16S-seq. In contrast, the abundances of 29 OTUs, which belong to different families including *Burkholderiaceae* (10 OTUs), *Fibrobacteraceae* (4 OTUs), *Paludibacteraceae* (5 OTUs), and *Micromonosporaceae* (3 OTUs), were significantly increased by 1.2–2.2 times in Cas-16S-seq compared with 16S-seq (Fig. 5d; Additional file 4: Table S6, Wald test, *p* < 0.01). This is likely because Cas-16S-seq obtained more bacterial sequences than 16S-seq. We also compared the composition of bacterial communities at the phylum level and found no significant difference between Cas-16S-seq and 16S-seq data in the relative abundance of predominant phyla (Fig. 5e). In summary, Cas-16S-seq efficiently removed targeted host 16S rRNA gene and enriched bacterial 16S rRNA genes from root samples.

### Simultaneous removal of chloroplast and mitochondrion 16S rRNA genes using multiplexed Cas-16S-seq

**Fig. 6:**
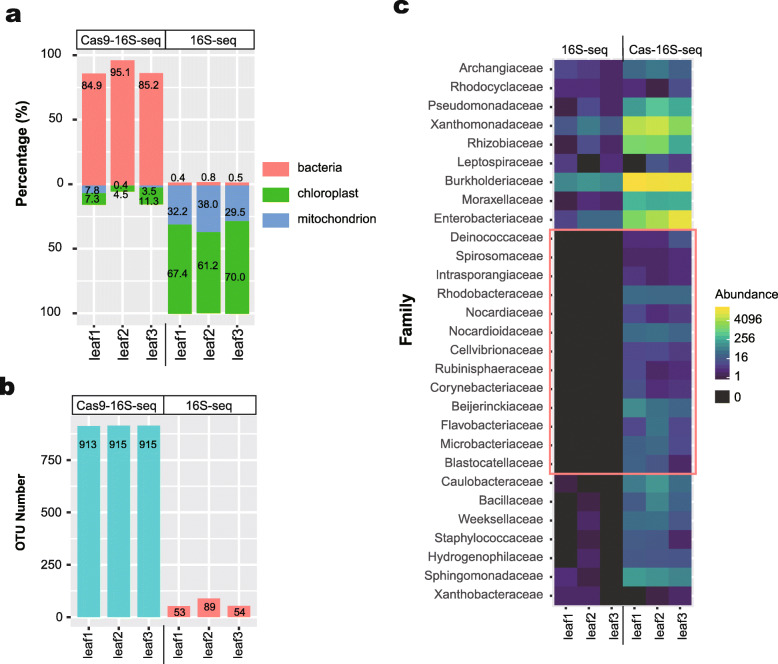
Simultaneous depletion of host mitochondrial and chloroplast 16S rRNA genes in phyllosphere samples using Cas-16S-seq. Two gRNAs (mt-gRNA780 and cp-gRNA578) targeting 515F-806R amplicon (V4 region) were used in Cas-16S-seq for three phyllosphere samples (leaf 1–3). The OTU table is rarefied to 50,000 reads (see also Additional file 5: Table S7 and S8). **a** Fractions of bacterial, mitochondrial, and chloroplast 16S rRNA genes in phyllosphere samples. **b** Comparison of OTU number with median abundance > 1 in each phyllosphere sample (also see Additional file 5: Table S7). **c** Comparison of bacterial community structure at family level (Cas9-16S-seq vs regular 16S-seq). The heatmap shows the abundances of bacterial families in each phyllosphere sample. The red box labels the bacterial families which are not observed in regular 16S-seq library

Our Cas-16S-seq with an mt-gRNA showed an efficient removal of rice mitochondrial 16S rRNA gene contamination in microbiome profiling. To further confirm the high applicability of our Cas-16S-seq, we also tested the efficiency and specificity of Cas-16S-seq to simultaneously remove mitochondrion and chloroplast 16S rRNA genes. To this end, we selected mt-gRNA780 and cp-gRNA578 to target rice 515F-806R amplicons in soil and phyllosphere samples. After quality filtration and denoising, we obtained a total of 580,666 sequences and then subsampled to the same sequencing depth (Additional file 5: Table S7). In soil samples, we detected almost identical bacterial OTUs with similar abundances in Cas-16S-seq and regular 16S-seq (Fig. 4c, d), indicating that Cas-16S-seq with mt-gRNA780 and cp-gRNA578 did not eliminate soil bacterial sequences (no bias) and produced the almost identical result with 16S-seq. In contrast, the fractions of host 16S rRNA genes were reduced from 99.4 ± 0.2% to 11.6 ± 5.9% in Cas-16S-seq of phyllosphere samples (Fig. 6a). This drastically increased the power to detect bacterial OTUs in phyllosphere samples. On average, 16S-seq only obtained 65 OTUs (median abundance > 1) from 288 bacterial sequences in phyllosphere samples, while Cas-16S-seq obtained 914 OTUs from 44,216 bacterial sequences (Fig. 6b, Additional file 5: Table S7 and S8, Student’s *t* test, *p* = 1.8e−4). As a result, Cas-16S-seq produced significantly enriched bacterial communities in comparison with 16S-seq (PERMANOVA, *R*^*2*^ = 0.634, *F* = 6.9, *p* = 0.017; Additional file 1: Figure S6). We examined the bacterial compositions at the family level and found that Cas-16S-seq detected 29 bacterial families but 16S-seq only detected 16 families (Fig. 6c). Like our previous analysis of 799F-1193R amplicons, we did not detect any bacterial OTUs whose relative abundance was significantly lower in Cas-16S-seq compared with 16S-seq in soil and phyllosphere samples, indicating that cp-gRNA578 and mt-gRNA780 did not off-target bacterial sequences in Cas-16S-seq. Together, Cas-16S-seq can efficiently and specifically remove both mitochondrion and chloroplast 16S rRNA genes in one reaction without off-targeting bacterial sequences, thereby greatly increasing the detection sensitivity of bacterial 16S metagenomics in plant microbiota samples.

## Discussion

In this study, we developed a Cas-16S-seq method that mitigates the contamination of 16S rRNA genes from host organelles in amplicon libraries. We established a bioinformatic pipeline to design gRNAs that specifically target host 16S rRNA gene with negligible off-targets on prokaryotic 16S rRNA genes (Fig. 2). As a proof of concept, five highly specific gRNAs, mt-gRNA1048, mt-gRNA1171, mt-gRNA1196, mt-gRNA780, and cp-gRNA578 were used to target the rice 799F-1193R and 515F-806R amplicons for Cas-16S-seq. After comprehensive comparisons of Cas-16S-seq and regular 16S-seq data using mock communities, paddy soil, rice root, and phyllosphere samples, we demonstrated that Cas-16S-seq drastically reduced the host 16S rRNA fraction but did not introduce bias to microbiota profiles. Thus, we anticipate that Cas-16S-seq would be a useful tool for plant microbiomics.

16S-seq is a common but powerful approach for studying the microbiome [6]. As an inherent problem, co-amplification of mitochondrial and plastid 16S rRNA genes by universal primers not only reduces the sensitivity and performance of 16S-seq but also increases the sequencing cost to study the plant microbiome. By engineering RNA-programmable Cas9 nuclease in 16S-seq, we developed the Cas-16S-seq method to remove host contamination for profiling the microbiome of plant samples. The major advantages of Cas-16S-seq are summarized as follows. First, Cas-16S-seq is highly efficient and robust in removing the target. Our data demonstrated that Cas-16S-seq using either gRNA can reduce the percentage of host fraction from 63.2 to 2.9% in root samples and from 99.4 to 11.6% in leaf samples, respectively (Fig. 5a and Fig. 6a). Cas-16S-seq also showed a better performance than the PNA PCR clamping method, particularly when the amount of bacterial DNA was lower than 20% (Fig. 3). Second, the additional cost for Cas9 and gRNA used in Cas-16S-seq is negligible. According to our experience, the cost of Cas9 and gRNA to process one sample is 8 Chinese Yuan (~ $1.15 US dollars) and Cas-16S-seq only requires an extra enzyme digestion step that needs less than 6 h. Because the abundant host 16S rRNA gene fraction was removed by Cas9 digestion, more indexed amplicons of Cas-16S-seq could be combined in one sequencing run. As a result, the overall cost per sample is reduced using Cas-16S-seq. Third, Cas-16S-seq is easily integrated into current 16S rRNA gene-based amplicon sequencing workflows. Only a Cas9/gRNA digestion step is added to standard 16S-seq library preparation. The Cas9 enzyme and gRNAs are commercially available or can be readily prepared in the laboratory. Host-specific gRNA could be designed using the method presented here or using other popular CRISPR gRNA design tools. Thus, it is easy to apply Cas-16S-seq in practice. Finally, Cas9 could be programmed to eliminate other targets in metagenomics in addition to mitochondrial and chloroplast 16S rRNA genes. For example, Cas9 could be used to mitigate host contamination in internal transcribed spacer-sequencing (ITS-seq) as we did for 16S-seq in this study. Importantly, Cas9 can simultaneously target different sites using multiple gRNAs. Therefore, mitochondrial and chloroplast 16S rRNA genes as well as other unwanted sequences could be cleaved and removed in one reaction using multiplexed Cas-16S-seq.

The host-specific gRNA is critical for Cas-16S-seq. The design of gRNAs for Cas-16S-seq is more challenging than other CRISPR/Cas9 applications because the gRNA is expected to discriminate one target from numerous homologs. Here, we adapted sophisticated bioinformatics programs, which we previously used in CRISPR-PLANT [42], to identify bacterial targets of mt-gRNA and cp-gRNA in the RDP-rRNA database (Fig. 2). We validated five highly specific gRNAs, and indeed, these gRNAs did not affect bacterial community structure in soil, rice root, and phyllosphere samples (Figs. 4, 5 and 6); even though mt-gRNA1048, mt-gRNA780, and cp-gRNA578 have few predicted RDP-rRNA off-targets (Additional file 1: Table S1 and S2). Using the same procedure, host-specific gRNAs could be designed for other plant species. In addition to CRISPR-PLANT, there are many bioinformatic tools for CRISPR gRNA design [46], and these tools can also be adapted to evaluate gRNA specificities for Cas-16S-seq. It should be noted that the specificities of mt-gRNA and cp-gRNAs are likely underestimated in this study using the RDP-rRNA database as a reference. This is because the bacterial 16S rRNA genes in the analyzed sample are only a small fraction of the RDP-rRNA dataset. For example, we detected 13,452 OTUs (abundance per sample > 10) in soil and root samples, which is only 0.4% of the RDP-rRNA data. However, the design of host-specific gRNA is based on known 16S rRNA genes, and the off-target risk to uncharacterized bacteria should be considered in Cas-16-Seq. Therefore, validating the specificity of selected gRNAs as we did in this study is recommended. In the future, the design of gRNA for Cas-16S-seq could be further refined using a more specific reference dataset according to sample type.

Similar to Cas9-mediated genome editing, Cas-16S-seq is potentially restricted by the off-targeting effect and PAM requirement of CRISPR/Cas systems. Indeed, the number of host-specific cp-gRNAs was restricted by these two factors in our analysis (Fig. 2d; Additional file 2: Table S2). The limited number of PAMs in host 16S rRNA gene and Cas9/gRNA recognizing imperfectly matched sequences greatly increases the chance of gRNA to have off-target sequences on RDP-rRNA dataset. Due to the fast evolution of CRISPR/Cas9 tools in genome editing, many Cas9 variants and homologs could be used to augment the targeting sites for Cas-16S-seq. In particular, the high fidelity Cas9 nuclease, which tolerates zero mismatches between the gRNA guide and DNA target [47], is expected to greatly increase the number of specific gRNAs for Cas-16S-seq. In addition, new Cas9 variants recognizing altered PAM sequences would expand the targetable sites in rice organelle 16S rRNA genes. If Cas9-NG, which recognizes a simple 5′-NG-3′ PAM [48], was used in Cas-16S-seq, over 800 gRNAs could be used to target rice chloroplast 16S rRNA gene. Moreover, many CRISPR/Cas systems have been characterized and engineered for DNA targeting in addition to Cas9 from *Streptococcus pyogenes*. These Cas nucleases recognize different PAMs, and some use gRNA with longer guide sequences than Cas9. For example, Cas12a recognizes a 5′-VTTT-3′ (*V* = A/G/C) PAM and its guide sequence is 24 nt long, which not only expands the targetable sites but also provides increased specificities in DNA targeting [49]. Applying these Cas nucleases in Cas-16S-seq would further enhance its ability to analyze plant microbial communities.

## Conclusions

We developed the Cas-16S-seq method that removes abundant host contamination using CRISPR/Cas9 for 16S rRNA gene-based amplicon sequencing. Our results showed that Cas-16S-seq is robust, effective, and low-cost, and importantly, does not introduce a bias when profiling plant microbial communities. This study also established a framework to use CRISPR/Cas9 to eliminate a target from millions of homologs in metagenomics.

## Methods

### Analysis of gRNA specificity

To design host-specific gRNAs that have minimal off-target effects on bacterial 16S rRNA genes, the CRISPR-PLANT bioinformatics pipeline, which we previously used to design highly specific gRNAs for genome editing, was modified to compare rice and bacterial 16S rRNA gene sequences (Fig. 2b) [50]. Rice chloroplast and mitochondrial 16S rRNA gene sequences were downloaded from the reference genome of *Oryza sativa L.* ssp *geng* cultivar Nipponbare (https://www.ncbi.nlm.nih.gov/genome/10). All Cas9-targetable 20 bp sequences preceding PAM (5′-NGG-3′) in rice mitochondrial and chloroplast 16S rRNA genes were extracted as guide sequences for mt-gRNA and cp-gRNA. To identify bacterial off-targets of mt-gRNA and cp-gRNA, a collection of 3,356,809 prokaryotic 16S rRNA gene sequences in the Ribosomal Database Project (RDP release 11, update 5, https://rdp.cme.msu.edu/, release 11) [39] was used as a reference for analysis. We extracted the 20 bp sequences before the 5′-NGG-3′ and 5′-NAG-3′ PAMs from the RDP-rRNA dataset. Then, pair-wise global alignment was performed between gRNA guide sequences and RDP-rRNA datasets using the VSEARCH program (version 2.8.1) [43, 51]. The VSEARCH program was run using the following setting: vsearch --usearch_global query-file -db RDP-guides --id 0.6 --strand plus --iddef 4 --minseqlength 1 --minwordmatches 1 --maxrejects 0 --maxaccepts 0 --samout output-file. Then, the RDP-rRNA off-targets of cp-gRNA and mt-gRNA were identified according to following criteria: for the off-target with NGG PAM, the sequence alignment of gRNA and RDP-rRNA (1) contained less than 4 mismatch/deletion/insertion (M/D/I) or (2) contained 4 M/D/I out of the seed region (12 bp next to the PAM); for the off-target with NAG PAM, the alignment between gRNA and RDP-rDNA (1) contained less than 2 M/D/I or (2) contained 2 M/D/I out of seed region (12 bp next to the PAM, Fig. 2a, b). For all off-target, no mismatch/gap is allowed at the last nucleotide of gRNA guide sequence (the nucleotide next to PAM) in alignment. Finally, the total number of RDP-rRNA off-targets for each gRNA was counted and used to rank the gRNA specificities. We removed 71,940 chloroplast sequences in RDP-rRNA dataset during analyzing cp-gRNAs. The analysis results are shown in Additional file 2: Table S1 and S2.

### In vitro digestion using Cas9 and gRNA

A plasmid vector (pUC19-gRNA, Addgene plasmid # 137776) containing the T7 promoter and gRNA scaffold was used for the in vitro transcription of gRNAs. The gRNA is cloned as described previously in Xie et al. [52]. For each gRNA, a pair of DNA oligos with appropriate overhangs were synthesized (Sangon Biotech, Shanghai, China) and annealed to dsDNA oligos. The oligo duplex was then ligated to the *Bsa*I-digested pUC19-gRNA vector, resulting in T7 promoter::gRNA constructs. The PCR fragment of the T7 promoter::gRNA cassette from the construct was used as a template for in vitro transcription using the HiScribe Quick T7 High Yield RNA Synthesis Kit (New England Biolabs, USA) following the manufacturer’s instructions. The synthesized gRNA was treated with DNase I (New England Biolabs, USA) and then purified with the RNA Clean & Concentrator Kit (Zymo Research, USA). The DNA oligo sequences for gRNA synthesis are shown in Additional file 6: Table S9.

Cas9/gRNA digestion was performed according to a previous report [22]. At the beginning, Cas9 (New England Biolabs, USA) and heat-denatured gRNA were assembled by incubating at 25 °C for 10 min. The DNA digestion was performed at 37 °C in a 30 μl reaction containing 30 nM Cas9, 30 nM gRNA, 100 ng of DNA substrate, and 1× NEBuffer3.1 (New England Biolabs, USA). Finally, the reaction was treated with 0.8 U of Protease K (New England Biolabs, USA) at 37 °C for 10 min and then purified with a QIAquick PCR Purification Kit (Qiagen, USA).

### Testing Cas-16S-seq using mock samples

To prepare mock samples, 799F-1193R fragments were amplified from rice mitochondrial and soil bacterial 16S rRNA genes and cloned into the pEASY-Blunt vector (TransGen Biotech, China) and confirmed by Sanger sequencing. Then, the 799F-1193R fragments were amplified from the plasmid using the primers M13F and M13R. Mock samples were prepared by mixing two PCR products at different ratios, resulting in mock communities containing 0%, 0.1%, 0.2%, 20%, 40%, 50%, and 100% bacterial 16S rRNA gene fragment. To evaluate Cas-16S-seq efficiency, mock samples were digested with Cas9/gRNA as described before. The digested product was amplified using primers M13F and M13R and analyzed by gel electrophoresis. To test the efficiency of the PNA PCR clamping method, a PNA oligo (5′-CCCCTGATCCGCGTAGA-3′) was synthesized by KareBay Biochem (USA). PCR clamping was performed by adding PNA oligo at a final concentration of 4 μM to PCRs. Controls without Cas9/gRNA digestion or PNA oligo were performed in parallel for all reactions.

### Collection of soil, rice root, and phyllosphere samples

The soil samples were collected from the rice field at the campus of Huazhong Agricultural University, Wuhan, China. Three biological replicates were collected for each gRNA. The rice root and phyllosphere samples were collected from rice plants at the tillering stage according to a previous report [29]. The soil particles were removed from the root by thoroughly washing with distilled water. The roots and leaves were washed 3 times with phosphate-buffered saline containing 0.1% TWEEN 20 and frozen in liquid nitrogen. Of note, these root samples include the rhizoplane and endosphere compartments. The genomic DNA of soil and plant samples was extracted using the Fast DNA SPIN Kit (MP Biomedicals, USA) following the manufacturer’s instructions. Negative controls were always included to monitor the contamination during sample collection and handling.

### Preparation of Cas-16S-seq amplicon libraries

The 16S-seq library was prepared using a two-step PCR procedure with the Bacterial 16S rDNA PCR Kit Fast (TaKaRa, Japan). In the first PCR, two universal primer pairs (Rd1 + 799F and Rd2 + 1193R, Rd1+515F and Rd2 + 806R) were used to amplify the V5-V6-V7 and V4 regions, respectively. The touchdown PCR condition was as follows: 94 °C for 3 min; 34 cycles of 94 °C for 1 min, annealing for 1 min, 72 °C for 45 s; and 72 °C for 10 min. The annealing temperature was set to 60 °C for 4 cycles, 58 °C for 6 cycles, 56 °C for 8 cycles, 54 °C for 8 cycles, and 52 °C for 8 cycles. After purifying the PCR products with Agencourt AMPure XP beads (Beckman Coulter, USA), 60 ng of amplicon was digested using Cas9 and gRNA as described above. The digestion was purified using chloroform/phenol extraction and ethanol precipitation. Then, a second index PCR was performed in a 25-μl reaction containing one third of the purified digestion as a template, 12.5 μl of I-5 2 × High-Fidelity Master Mix (Molecular Cloning Laboratories, USA), and 1.25 μl of P5-index-bc1 (10 μM) and P7-index-bc2 (10 μM) (see Additional file 6: Table S10 for primer sequences). The PCR program was as follows: initial denaturation at 98 °C for 1 min; 8 or 15 cycles of 98 °C for 30 s, 58 °C for 30 s, and 72 °C for 1 min; and final extension at 72 °C for 5 min. The amplified product was purified using Agencourt AMPure XP beads (Beckman Coulter, USA) and combined at an equal molar ratio. The mixed amplicons were sequenced with an Illumina HiSeq 2500 using 2 × 250 bp paired-end reads. For each step, controls were always included to monitor contamination from reagents, primers, and plastic consumables. All primers were synthesized by Sangon Biotech (China), and their sequences are shown in Additional file 6: Table S10.

### Bioinformatics analysis of 16S-seq data

The raw sequences’ processing and OTU clustering were performed using the VSEARCH pipeline [51]. After demultiplexing and merging the paired-end reads, primers and an extra 20 bp at the 3′-end were trimmed using Cutadapt (version 2.5) [53]. We estimated the maximum expected error (maxEE) for each sequence and discarded sequences with maxEE values higher than 1 [51]. Chimeras were detected and removed at the sample level and whole study level [54]. Finally, the denoised sequences were clustered to OTUs at 100% identity. The taxonomy of each OTU was assigned using the DADA2 (version 1.10.1) assignTaxonomy function with 60 bootstraps [55], and the SILVA training set (version 132) was used as a reference for taxonomy assignment [40, 55].

### Statistical analyses

For each experiment, the OTU table was rarefied to the same sequence number. The statistical significance was determined at *α* = 0.05, and analyses were performed using R packages (version 3.5.1). Bacterial diversities were analyzed using the Phyloseq package (version 1.26.1) in R after removing OTUs related to 16S rRNA genes from rice and normalizing OTU abundances by rarefaction [56]. For soil samples (Fig. 4; Additional files 3 and 5), we rarefied OTU table to 16,000 sequences per sample and used paired Student’s *t* test to compare the bacterial diversities (OTU number, Shannon and Simpson indices) between Cas-16S-seq and regular 16S-seq results. For root samples (Fig. 5; Additional file 4), we calculated the relative abundance of rice mitochondrial 16S rRNA gene sequences per sample per gRNA in the normalized dataset which was rarefied to 41,500 sequences. The data was subject to ANOVA to test the effects of Cas9/gRNA digestion, gRNA types, and PCR cycle number on bacterial diversities (OTU number, Shannon and Simpson indices). For phyllosphere samples (Fig. 6; Additional file 5), the OTU table is rarefied to 50,000 sequences and Student’s *t* test was used to compare the difference of OTU number between Cas-16S-seq and regular 16S-seq.

To identify differential OTUs between Cas-16S-seq and regular 16S-seq in each experiment, DESeq2 (version 1.22.2) [45] was used to identify differential OTUs between regular 16S-seq and Cas-16S-seq for each gRNA. The Wald test in DESeq2 was performed to test differences in OTU relative abundances. We also performed permutational multivariable analysis of variance (PERMANOVA) based on Bray-Curtis dissimilarity to estimate the effects of Cas9 digestion to bacterial community composition. PERMANOVA was conducted using the adonis function in Vegan package (version 2.5-6) [57].

## Supplementary information

**Additional file 1: Figure S1.** Sequence alignment of 16S rRNA genes from rice mitochondrial (Mito), chloroplast (Chlo) and *E. coli* (*rrsA*). The variable regions (V1-V9) are labeled with different colors. The positions of universal primers are shown in alignments. The codes for degenerate nucleotides in the universal primer sequence are R = G/A, Y = T/C, K = G/T, M = A/C, S = G/C, D = G/A/T, H = A/C/T, N = A/T/G/C. **Figure S2.** Amplification of rice mitochondrial and/or plastid 16S rRNA gene using four universal primer pairs. The rice genomic DNA template was prepared from seedlings grown in sterilized Murashige and Skoog medium. The PCR products of 27F-338R, 515F-806R, 799F-1193R and 1114F-1392R were separated by gel electrophoresis and confirmed by Sanger sequencing. The 27F-338R product only contains amplicons of chloroplast 16S rRNA gene; the 515F-806R products include amplicons of chloroplast and mitochondrial 16S rRNA genes; the 799F-1193R product only contains mitochondrial 16S rRNA gene fragment; and the 1114F-1392R product only contains chloroplast 16S rRNA fragment. NC, negative control using distilled water as a template. **Figure S3.***In vitro* DNA cleavage activity of Cas9 with 12 mt-gRNAs and 5 cp-gRNAs. The purified rice amplicon was used as the substrate. The number at the bottom of each lane indicates the digestion efficiencies estimated from the intensities of cleaved bands using Image J (https://imagej.nih.gov/ij/). **Figure S4.** Gel electrophoresis of Cas-16S-seq (Cas9+) and regular 16S-seq (Cas9-) amplicons of rice root samples. #1-#3 indicate three biological replicates; * indicate the rice mitochondrial 799F-1193R amplicons. **Figure S5.** Percentages of chimeric sequences in amplicons of root samples. The chimeras were identified using the VSEARCH –uchime_denovo function with default setting. Statistical differences between Cas-16S-seq and regular 16S-seq (CK) were tested using ANOVA. **Figure S6.** PERMANOVA analysis results.

**Additional file 2: Table S1.** Result of mt-gRNA specificity prediction. **Table S2**. Result of cp-gRNA specificity prediction.

**Additional file 3: Table S3.** Summary of soil 799F-1193R amplicon data. **Table S4.** Differential OTUs in soil samples (Cas-16S-seq vs regular 16S-seq).

**Additional file 4: Table S5.** Summary of root 799F-1193R amplicon data. **Table S6.** Differential OTUs in root samples (Cas-16S-seq vs regular 16S-seq).

**Additional file 5: Table S7.** Summary of 515F-806R amplicon result. **Table S8.** Abundance of bacterial OTUs detected in leaf 515F-806R amplicons.

**Additional file 6: Table S9.** Primers used for *in vitro* transcription of gRNA. **Table S10.** Primer oligos used for 16S rRNA gene amplification.

## Data Availability

The microbiome metagenomics data used in this study are available at the Genome Sequence Archive in BIG Data Center [58], Beijing Institute of Genomics (BIG), Chinese Academy of Sciences, under the accession number (PRJCA001629) [59]. The codes used in this study are stored at GitHub [60]. The plasmid vectors pUC19-gRNA is deposited in Addgene (plasmid # 137776).
