# Supplementary material for: Engineering CRISPR/Cas9 to mitigate abundant host contamination for 16S rRNA gene-based amplicon sequencing
Source: Microbiome. 2020 Jun 3;8:80. doi: 10.1186/s40168-020-00859-0 (PMC7268715; doi:10.1186/s40168-020-00859-0)
Supplement: Supplementary file 2 — Additional file 1: Figure S1. Sequence alignment of 16S rRNA genes from rice mitochondrial (Mito), chloroplast (Chlo) and E. coli (rrsA). The variable regions (V1-V9) are labeled with different colors. The positions of universal primers are shown in alignments. The codes for degenerate nucleotides in the universal primer sequence are R = G/A, Y = T/C, K = G/T, M = A/C, S = G/C, D = G/A/T, H = A/C/T, N = A/T/G/C. Figure S2. Amplification of rice mitochondrial and/or plastid 16S rRNA gene using four universal primer pairs. The rice genomic DNA template was prepared from seedlings grown in sterilized Murashige and Skoog medium. The PCR products of 27F-338R, 515F-806R, 799F-1193R and 1114F-1392R were separated by gel electrophoresis and confirmed by Sanger sequencing. The 27F-338R product only contains amplicons of chloroplast 16S rRNA gene; the 515F-806R products include amplicons of chloroplast and mitochondrial 16S rRNA genes; the 799F-1193R product only contains mitochondrial 16S rRNA gene fragment; and the 1114F-1392R product only contains chloroplast 16S rRNA fragment. NC, negative control using distilled water as a template. Figure S3.In vitro DNA cleavage activity of Cas9 with 12 mt-gRNAs and 5 cp-gRNAs. The purified rice amplicon was used as the substrate. The number at the bottom of each lane indicates the digestion efficiencies estimated from the intensities of cleaved bands using Image J (https://imagej.nih.gov/ij/). Figure S4. Gel electrophoresis of Cas-16S-seq (Cas9+) and regular 16S-seq (Cas9-) amplicons of rice root samples. #1-#3 indicate three biological replicates; * indicate the rice mitochondrial 799F-1193R amplicons. Figure S5. Percentages of chimeric sequences in amplicons of root samples. The chimeras were identified using the VSEARCH –uchime_denovo function with default setting. Statistical differences between Cas-16S-seq and regular 16S-seq (CK) were tested using ANOVA. Figure S6. PERMANOVA analysis results. [file 40168_2020_859_MOESM1_ESM.pdf]

Supplementary Figures S1-S6

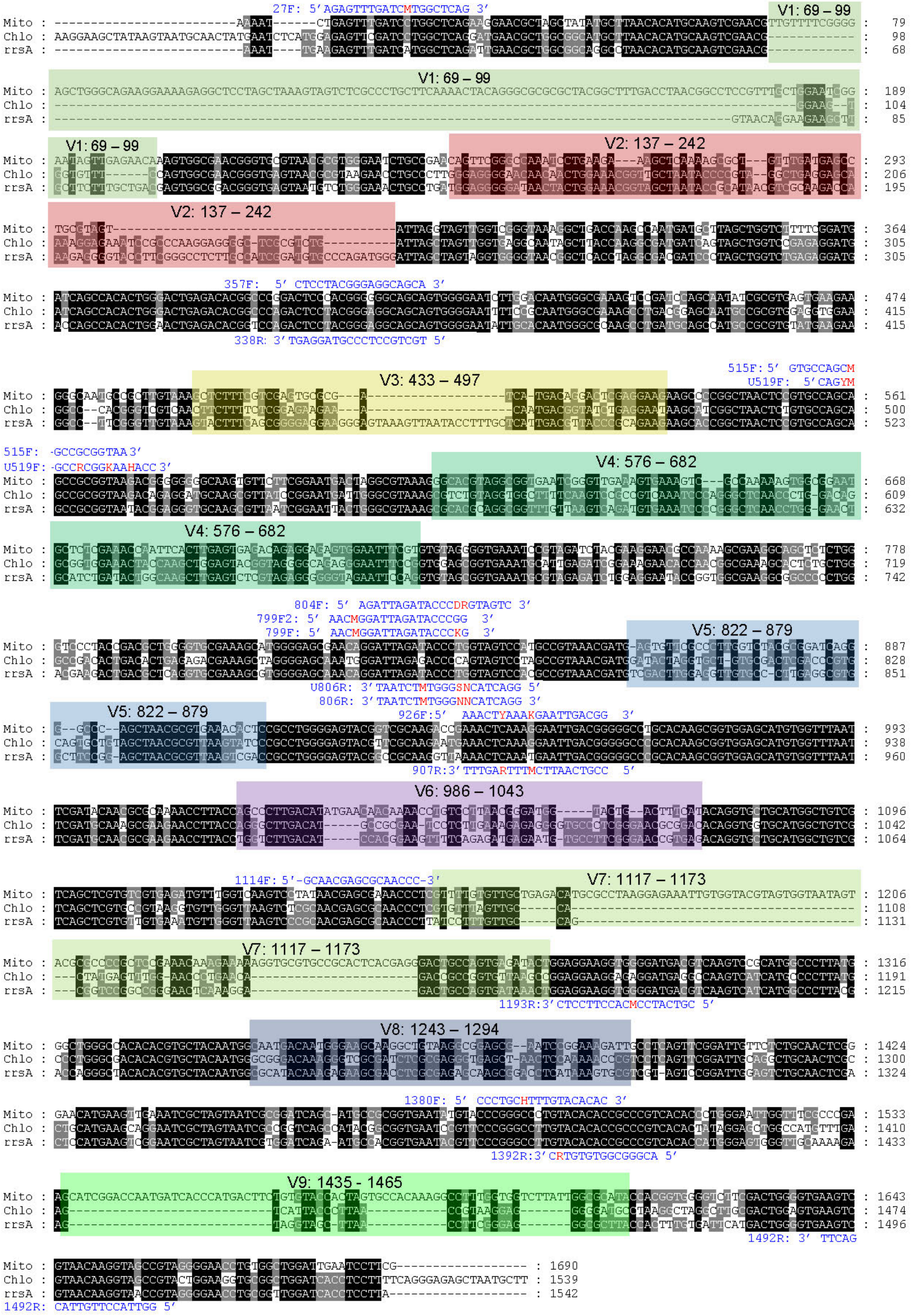

Figure S1. Sequence alignment of 16S rRNA genes from rice mitochondrial (Mito), chloroplast (Chlo) and *E. coli* (*rrsA*). The variable regions (V1-V9) are labeled with different colors. The positions of universal primers are shown in alignments. The codes

for degenerate nucleotides in the universal primer sequence are R=G/A, Y=T/C, K=G/T, M=A/C, S= G/C, D=G/A/T, H= A/C/T, N=A/T/G/C.

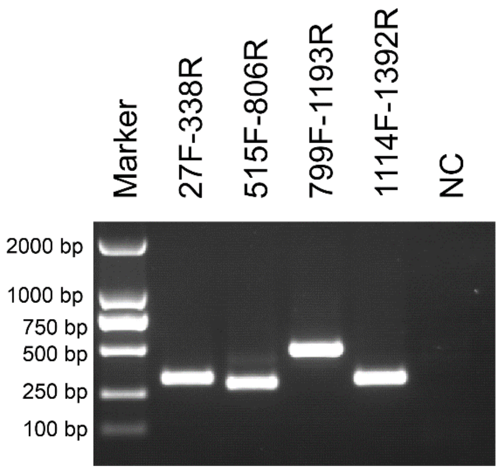

Figure S2. Amplification of rice mitochondrial and/or plastid 16S rRNA gene using four universal primer pairs. The rice genomic DNA template was prepared from seedlings grown in sterilized Murashige and Skoog medium. The PCR products of 27F-338R, 515F-806R, 799F-1193R and 1114F-1392R were separated by gel electrophoresis and confirmed by Sanger sequencing. The 27F-338R product only contains amplicons of chloroplast 16S rRNA gene; the 515F-806R products include amplicons of chloroplast and mitochondrial 16S rRNA genes; the 799F-1193R product only contains mitochondrial 16S rRNA gene fragment; and the 1114F-1392R product only contains chloroplast 16S rRNA fragment. NC, negative control using distilled water as a template.

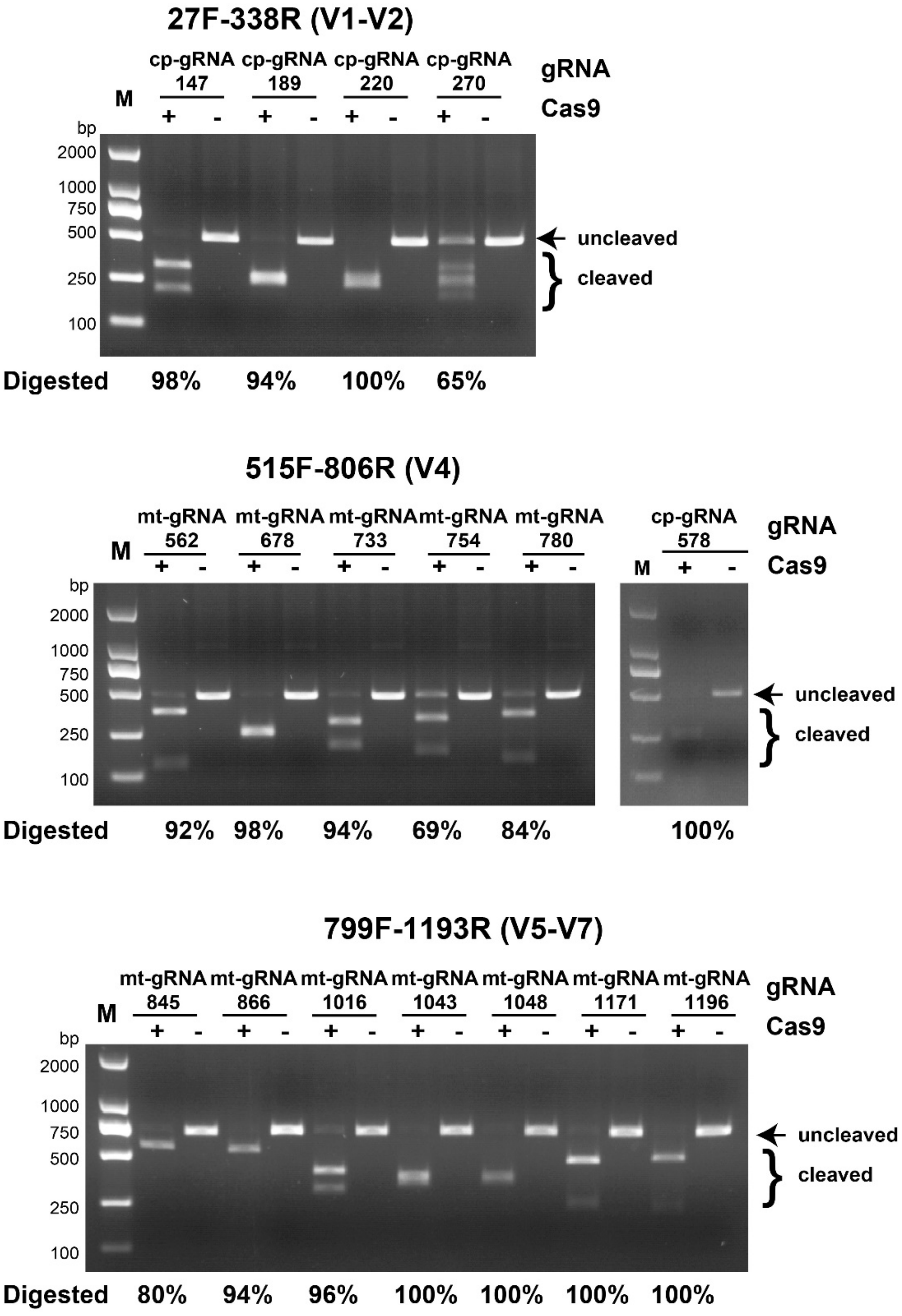

Figure S3. ***In vitro*** **DNA cleavage activity of Cas9 with 12 mt-gRNAs and 5 cp-gRNAs.** The purified rice amplicon was used as the substrate. The number at the bottom of each lane indicates the digestion efficiencies estimated from the intensities of cleaved bands using Image J (https://imagej.nih.gov/ij/).

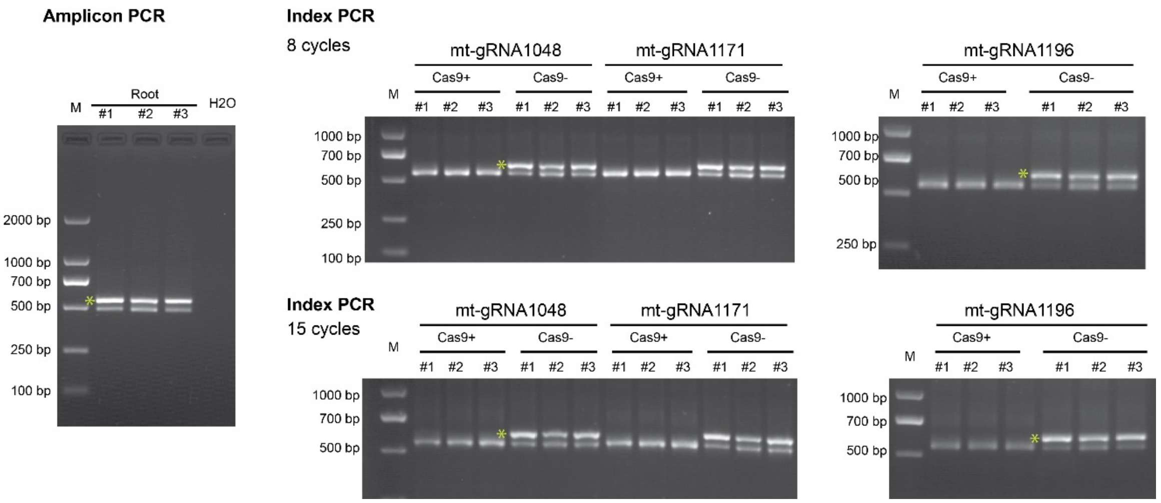

Figure S4. Gel electrophoresis of Cas-16S-seq (Cas9+) and regular 16S-seq (Cas9-) amplicons of rice root samples. #1-#3 indicate three biological replicates, * indicate the rice mitochondrial 799F-1193R amplicons.

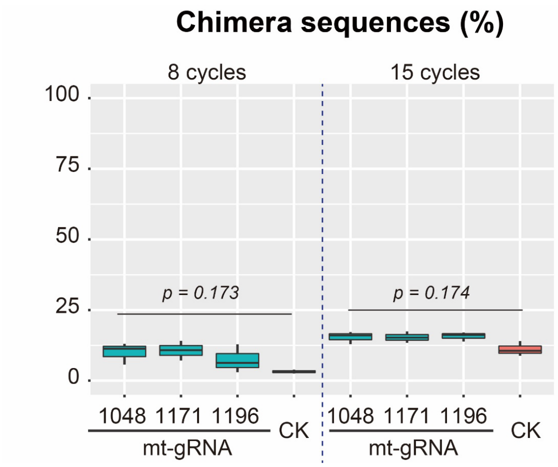

gRNA	Cycle_No	Chimera (%, mean+sd)
gRNA1048	8	13.2%±5.3%
gRNA1171	8	14.1%±4.7%
gRNA1196	8	10.0%±7.6%
CK	8	4.4%±0.9%
gRNA1048	15	20.1%±2.5%
gRNA1171	15	20.2%±2.5%
gRNA1196	15	20.3%±2.3%
CK	15	15.7%±3.2%

Figure S5. Percentages of chimeric sequences in amplicons of root samples. The chimeras were identified using the VSEARCH –uchime_denovo function with default setting. Statistical differences between Cas-16S-seq and regular 16S-seq (CK) were tested using ANOVA.

Root samples (799F-1193R)

	Df	Sum Of Sqs	R^2	F	Pr(>F)	Significance
Plant_sample	2	2.9096	0.67315	34.774	0.001	***
gRNA	3	0.6487	0.15009	5.169	0.001	***
Cycle_No	1	0.0528	0.01222	1.263	0.246	
Residual	17	0.7112	0.16454			
Total	23	4.3224	1			

Phyllosphere samples (515F-806R)

	Df	Sum Of Sqs	R^2	F	Pr(>F)	Significance
Plant_sample	2	0.3293	0.18293	1.0045	0.61667	
Cas9	1	1.14301	0.63496	6.9732	0.01667	*
Residual	2	0.32783	0.18211			
Total	5	1.80015	1			

Significant. codes: 0 ‘***’ 0.001 ‘**’ 0.01 ‘*’ 0.05 ‘.’ 0.1 ‘ ’ 1

Figure S6. PERMANOVA analysis results.
